# A hippocampal digital Twin reveals latent structure in freely flying bats

**DOI:** 10.64898/2026.10.09.758148

**Published:** 2026-10-13

**Authors:** Adam S. Lowet, Yongrong Qiu, Angelo Forli, Krishna Chaitanya Kasuba, Yang Lin, Andreas S. Tolias, Michael M. Yartsev

## Abstract

Concurrent advances in artificial intelligence (AI), large-scale neuronal recording, and behavioral tracking have enabled increasingly powerful predictive models of brain-behavior relationships. Yet predictive accuracy does not automatically yield interpretable principles of neural organization, particularly during natural behavior, where the relevant variables computed are often unknown in advance. Here, we developed a functional digital twin of the hippocampus, trained jointly on large-scale neural activity and self-directed three-dimensional flight in freely flying bats. A multi-modal, multitask transformer accurately predicted both neural activity and behavior, outperformed standard approaches, and generalized across recording sessions. Interrogating the model’s representations suggested a form of hippocampal generalization across self-selected trajectories that was not apparent in physical coordinates, but was plainly visible in the model’s low-dimensional latent space. We confirmed this organization directly in the recorded neural activity: neurons fired at corresponding stages of spatially distinct paths connecting the same origin and destination, preserving their relative firing order even as their firing fields shifted with the path. Analysis of neuron-specific readouts further identified functional relationships among neurons that generalized from flight to waking rest and sleep, brain states never used for training, and differed systematically between the hippocampal subregions CA1 and CA3. Together, these results show how AI-based functional digital twins can complement conventional analysis by generating testable hypotheses about neural organization during natural behavior.

